# THE IMPLICATIONS OF HEALTH FOR RETIREMENT DECISIONS FOR NURSES IN THE CZECH REPUBLIC, IRELAND, SWEDEN, AND THE UK

**DOI:** 10.1093/geroni/igae098.0975

**Published:** 2024-12-31

**Authors:** Aine Ni Leime, Margaret O’Neill, Marie Pospisilova, Jakov Jandric, Clary Krekula

**Affiliations:** University of Galway, Galway, Galway, Ireland; University of Galway, Galway, Galway, Ireland; Czech Academy of Sciences, Prague, Hlavni mesto Praha, Czech Republic; University of Edinburgh, Edinburgh, Scotland, United Kingdom; Linnaeus University, Marsta, Stockholms Lan, Sweden

## Abstract

This study examines the implications of health considerations for retirement decisions among older nurses in Ireland, Czechia, Sweden, and the UK, in the context of demographic ageing and extended working life policies. Employing a life course perspective, the concept of cumulative advantage/disadvantage, and a gendered political economy of aging framework, this internationally comparative study focuses on the impact of the physical and emotional demands of nursing and diverse national policies on nurses’ retirement decisions. Through analyzing data from qualitative interviews with 148 pre/retirement-age nurses in four European countries conducted as part of an international EU study, findings reveal significant health challenges, including musculoskeletal injuries and work-related stress, as central in the decision-making process. The study highlights gendered nuances in these decisions, with societal caring norms and financial constraints due to low pensions pressing many female nurses to extend their working lives or face precarious retirements. Despite the onerous physical demands of nursing and a general reluctance to work past 60, options like part-time work and job sharing emerge as critical for those considering extended work. This research highlights the misalignment of current policies with the lived experiences of older nurses, proposing reforms that recognize the gendered and health-related implications of the profession. Recommendations include re-evaluating extended working life policies with a gender-sensitive lens, introducing flexible employment options, ensuring choice in retirement timing, and providing provisions for early retirement due to health issues.

